# Electronic Nose: Recent Developments in Gas Sensing and Molecular Mechanisms of Graphene Detection and Other Materials

**DOI:** 10.3390/ma13010080

**Published:** 2019-12-22

**Authors:** Sylwia Orzechowska, Andrzej Mazurek, Renata Świsłocka, Włodzimierz Lewandowski

**Affiliations:** 1 M. Smoluchowski Institute of Physics, Jagiellonian University, 30-348 Krakow, Poland; 2 Faculty of Pharmacy, Medical University of Warsaw, 02-097 Warszawa, Poland; andmazurek@wp.pl; 3 Department of Chemistry, Biology and Biotechnology, Bialystok University of Technology, 15-351 Bialystok, Poland; r.swislocka@pb.edu.pl (R.Ś.); w-lewando@wp.pl (W.L.)

**Keywords:** graphene, electronic nose, carbon nanotubes, porphyrins, conductive polymers

## Abstract

The aim of the study was to present the possibility of the sensitivity improvement of the electronic nose (e-nose) and to summarize the detection mechanisms of trace gas concentrations. Our main area of interest is graphene, however, for the better understanding of the sensing mechanisms, it is crucial to review other sensors of similar functions. On the basis of our previous research, we explained the detection mechanism which may stay behind the graphene sensor’s sensitivity improvement. We proposed a qualitative interpretation of detection mechanisms in graphene based on the theory regarding the influence of metals and substituents on the electronic systems of carbon rings and heterocyclic aromatic ligands. The analysis of detection mechanisms suggests that an increase of the electronic density in graphene by attaching a substituent and stabilization of electronic charge distribution leads to the increase of graphene sensor conductivity. The complexation of porphyrins with selected metals stabilizes the electronic system and increases the sensitivity and selectivity of porphyrin-based sensors. Our research summary and proposed conclusions allow us to better understand the mechanisms of a radical change of graphene conductivity in the presence of trace amounts of various gases.

## 1. Introduction

Molecular mechanisms of detection of trace amounts of gases have been studied for many years. Moreover, different analyses and data interpretation methods have been developed over the past decades. These achievements allowed us to get more detailed insights into the concept of electronic nose (e-nose) which represents a method that is complementary to the commonly used GC-MS technique (gas chromatography combined with mass spectrometry). In contrast to GC-MS, the use of the e-nose does not allow for direct chemical identification of the particular substance, but it can show the final specific response of the sensors to the analyzed substance present in the sample and assign it to a specific group of compounds. Moreover, the e-nose method may allow for the production of small, inexpensive and user-friendly devices that can be used in different areas where gas identification mechanisms play a significant role (e.g., industry applications, healthcare, food and air quality control, etc.). 

An electronic nose is a model of the mammalian olfactory system. According to the Axel and Buck theory (Nobel Prize, 2004), the perception process begins in the olfactory epithelium, where approximately fifty million receptor neurons initially identify and classify volatile molecules [1]. Each of neurons is equipped in a dendrite ended in a bulb, from which cilia extend. G-protein coupled receptors, which were described by Robert Lefkowitz and Brian Kobilka (Nobel Prize in chemistry, 2012), are located on the surface of cilia and play the role of chemosensory receptors [2]. The scale of similarity of the molecule shape to the pattern assigned to the receptor corresponds to the intensity of the electric impulse. A single receptor is activated by many odors and a substance can be recognized by many receptors. The receptor, when a matching molecule is detected, begins transmission by triggering the opening of ion channels in neurons and depolarization of a cell membrane. The electric potential difference moves to the synapse and finally to the dendrites of postsynaptic neurons.

Currently, research is focused on applying the e-nose in medical fields, especially in the early diagnosis and prevention of respiratory diseases (i.e., lung cancer) using, among others, quartz microbubbles as well as detecting the presence of bacteria in the urine and the eyeball using polymer sensors [3]. The e-nose has also diagnostic capabilities for kidney, prostate, bladder, and even Parkinson’s disease [4,5,6]. This extremely interesting technology needs to be improved in terms of features related to price or size but undeniably it will be increasingly introduced into our daily life due to its reliability and advantages over conventional odor analysis methods [7]. 

**Figure 1:**
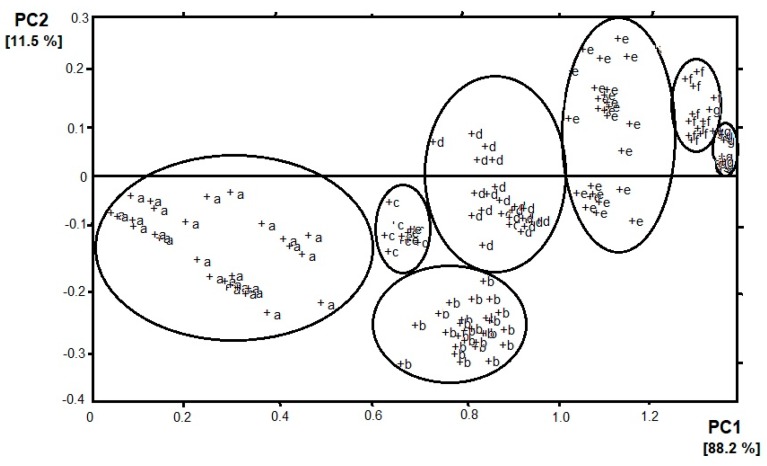
Result of principal component analysis (PCA) (PC1 vs. PC2) analysis of e-nose response on banana volatile compounds. Clusters were determined according to the increasing ripeness of the fruit (a–g).

The development of artificial olfaction methods would not be so dynamic without the implementation of different methods that allow for a detailed analysis of data sets of sensors arrays. Here, we would like to highlight the two most popular and useful methods—principal component analysis (PCA) and cluster analysis (CA). PCA is a dimensionality-reduction method that is often used to reduce the dimensionality of large data sets, by transforming a large set of variables into a smaller one that still contains most of the information from the large set. By applying dimensionality reduction, we trade a little accuracy for simplicity. The reason for this is that smaller data sets are easier to explore and visualize which makes analyzing data much easier and faster for machine learning algorithms without extraneous variables to process. Thanks to PCA analysis, sensors are assayed for their responsibility for the vapors classification. Sensors with loadings ~ 0, for a particular principal component, have a minor contribution to the total response of the array, whereas, high values show discriminating sensors. According to this theory, sensors that have an inconsiderable responsibility for the distribution pattern in the PCA plot are usually removed from the sensor array, because of a negative effect on the pattern resolution. Moreover, sensors with equal loading parameters can be presented by just one sensor [8]. In the case of e-nose, the n-dimensional response of the sensor matrix is often approximated by means of two odor components. This solution facilitates the interpretation of results, e.g., by imaging on a plane. An example is presented in Figure 1 where the result of the PCA analysis of e-nose (4 sensors) response on the presence of banana volatile compounds can be observed [9]. Four sensors are used because of the sensitivity to different volatiles (sensors 1 and 4—volatile organic compounds; sensor 2—hydrocarbons, sensor 3—carbon monoxide). The objective of PCA analysis is the establishment of categories for the state of banana ripeness. Two principal components are kept, which accounted for 99.7% of the variance in the data— PC 1 and PC 2 correspond to 88.2% and 11.5% of the variance, respectively. The seven categories of vapors appear which correspond to the seven states of ripeness. Most of the variance in the data is described by analyzing the two first principal components, which suggests that the sensor responses are strongly correlated. The loadings for PC 1 of sensors 1, 3, and 4 are rather similar but for PC 2 they are quite different. It can be assumed that the categories of vapors established by PCA analysis are consistent with there being different states of ripeness. Therefore, from left to right in Figure 1, the clusters appear ordered according to increasing ripeness. The clusters a, d, and e show a significant spread in a direction that is perpendicular to the direction of higher ripeness. The spread may suggest the drift in the sensor response or the changes in the state of fruit ripeness [10].

**Figure 2:**
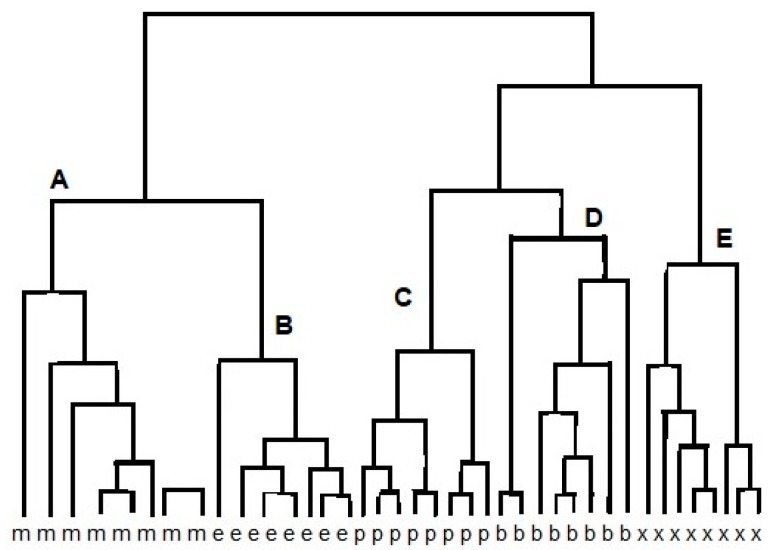
Result of cluster analysis (CA) of e-nose response on methanol (m), ethanol (e), propanol (p), butanol (b), and methyl-butanol (x), resulting in clusters A, B, C, D, E.

The cluster analysis (CA) is a class of methods that are applied to classify objects or cases into relative groups named clusters. CA may be used to the obtained data set. The reaction between different vapors and various reduced graphene oxide (RGO) sensors results in data points that are localized close to each other for similar sensors. Therefore, chemically similar sensors should be classified into one cluster [11]. Figure 2 shows an example of the result of using the CA method to analyze the response of e-nose (12 sensors) to alcohol samples: methanol, ethanol, propanol, butanol, and methyl-butanol. Grouping of points (clusters) can be done in many ways. Connecting points is one of grouping method. In the first connecting step there are n single-point clusters. At each step, we connect the closest ones and as a result, we obtain a connecting tree (dendrogram) [9]. The grouping is based on the proximity of the vectors in feature space. When the same array is presented to a set of few odors, the responses can be regarded as a set of few vectors, which are represented by a response matrix. In the response matrix, each column represents a response vector associated with a particular odor, whereas the rows are the responses of an individual sensor to the different measurands. As odor sensors are not entirely specific, an individual sensor will respond to a variety of odors but with varying sensitivity. CA reveals high correlations between the tin oxide sensors in the array. The correlation matrix of the conductance change for alcohol is calculated. Strong correlations exist between sensors (1, 3, 5, 6, 9, 11, 12) and (7, 8). Therefore, the vapors can be measured using a subset of only five sensors, such as 1, 2, 4, 7, and 10 [9,12].

Development of new analysis methods, improvement of sensitivity and selectivity of detectors as well as the better understanding of sensing mechanisms allow to popularize the use of the e-nose in the industry and medicine.

## 2. Sensing Mechanisms and Methods to Improve the Sensitivity of Carbon Nanotubes, Porphyrins, and Graphene Sensors

### 2.1. Sensors Based on Carbon Nanotubes

Carbon nanotubes (CNT) found widespread use as gas sensors due to their electronic properties and large surface area. A significant improvement in the sensitivity and selectivity of CNT-based sensors has been achieved by the formation of various types of hybrids. It was shown that the formation of a CNT hybrid with a metal oxide (e.g., SnO_2_) significantly improves the sensitivity of the detector and secures an excellent response to NO_2_ [13], CO [14], NH_3_ [15], and H_2_ [16]. Sensors, which are hybrids of CNT and metal oxides (MOX) can be divided into two types depending on the quantitative advantage of one of the components. The first type of sensor is a CNT hybrid with MOX attached to the sidewall of the nanotube. Formation of the CNT-MOX hybrid is possible due to the introduction of functional groups on the surface of the CNT which requires a CNT oxidation with a strong carboxylic acid. The second type of hybrid sensor is a matrix built of MOX and incorporated CNTs, while the method of implementation can be carried out according to four techniques described by Kerdcharoen et al. [17]. An important advantage of CNT-MOX complexes is a significant improvement in sensitivity and specificity, as well as a decrease in the sensor work temperature. The improvement of the above-mentioned parameters is attributed to the formation of the interfacial surface (heterojunctions) between the two coatings of different semiconductor crystals and the formation of nanochannels through which gas molecules are transported to the gas-sensitive layers [17].

Carboxylated single-wall carbon nanotubes (SWNT-COOH) found application in the detection of amines, ammonia, trimethylamine, and dimethylamine. An increase of the sensor sensitivity based on SWNT-COOH was achieved by forming an SWNT-COOH composite with polymers, such as polyvinyl chloride, polyvinylpyrrolidone, and cumene ending with polystyrene-co-maleic anhydrite [18,19]. The polymers used to form the composite are non-conductive, therefore, the role of conducting channels is played by nanotubes dispersed in a polymer matrix. Adsorption of the gas on the polymer matrix induces an increase of the space between the channels and changes in the material conductivity. The significant advantage of the SWNT-COOH-polymer composite is the ability to detect gases in a wide range of concentrations (50–1000 ppm) [18,20].

**Figure 3:**
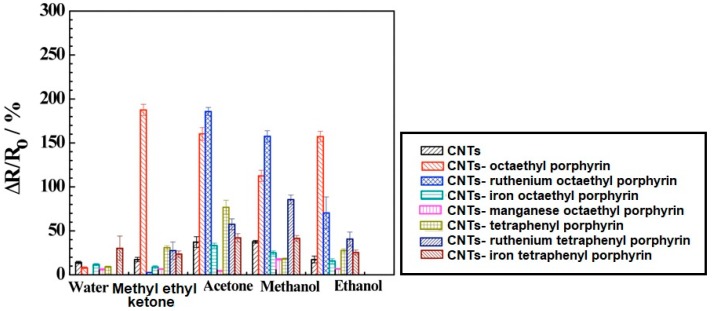
Comparison of carbon nanotubes (CNT) and various CNT-porphyrins hybrids responses towards different gases and water. Reprinted with the authors’ permission [21].

Despite the particular physicochemical properties of carbon nanotubes, the low sensitivity to volatile organic compounds is a serious limitation. The increase in the sensitivity of sensors based on carbon nanotubes was achieved by creating a CNT hybrid with porphyrins and metalloporphyrins. The formation of the hybrid is based on the reaction of the porphyrin core with the sidewall of the nanotube [21,22,23]. The core of the metalloporphyrin binds the fragrance molecules, thus, the metalloporphyrin is the donor system that changes the electronic charge distribution in the CNT. An increase in the negative charge in the CNT leads to an increase of the material conductivity. The sensing response of CNT-porphyrin hybrids can be assigned to electrostatic gating due to charge transfer and modification of the Schottky barrier which results in work function change and reduced charge mobility by the introduced scattering sites [21,24]. Figure 3 presents the histogram showing a comparison of CNT and various CNT-porphyrin hybrid responses to different gases and water. The ∆R/R_0_ was defined as ΔR/Ro% = (R − Ro)/Ro * 100, where R stands for the resistance of the device exposed to the analyte and Ro stands for the initial resistance before analyte exposure. It can be noticed that the conductance of bare CNTs decreased significantly upon functionalization with ruthenium complexes with porphyrin. Moreover, the ruthenium complexes with porphyrin-coated CNTs had a more negative threshold gate voltage and lower transconductance when compared to the bare CNTs. These changes are attributed to the n-doping by the electron donor porphyrin of the p-type semiconductor CNT which results in lower carrier (hole) concentration and carrier mobility [21].

**Figure 4:**
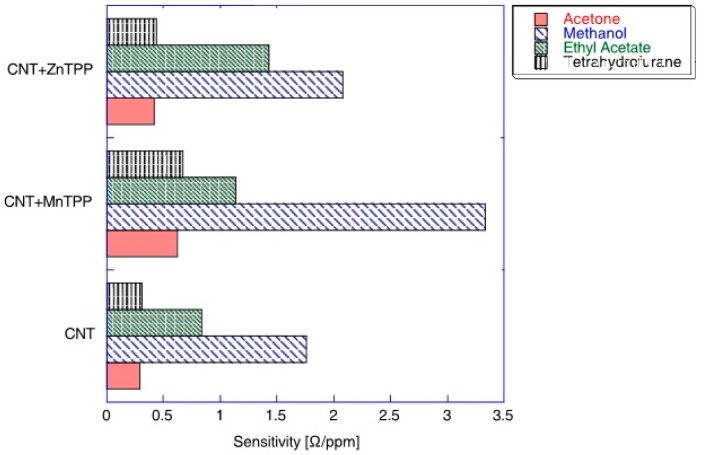
Sensitivity of the sensors composed of Mn-tetraphenylporphyrin-CNT, Zn-tetraphenylporphyrin-CNT hybrids, and CNT towards four vapors. The maximum sensitivity corresponds to the sensor sensitivity at low concentrations. Reprinted with the authors’ permission [25].

A comparison of the sensitivity of CNT-porphyrins hybrids towards acetone, methanol, ethyl acetate, and tetrahydrofurane is presented in Figure 4. The diagram confirms that porphyrin-based sensors are characterized by a large cross-selectivity, therefore, there is no evident advantage in comparison to bare CNT. Significantly higher sensitivity can be observed for methanol and ethyl acetate, while for acetone and tetrahydrofurane it is almost undistinguished [25].

**Figure 5:**
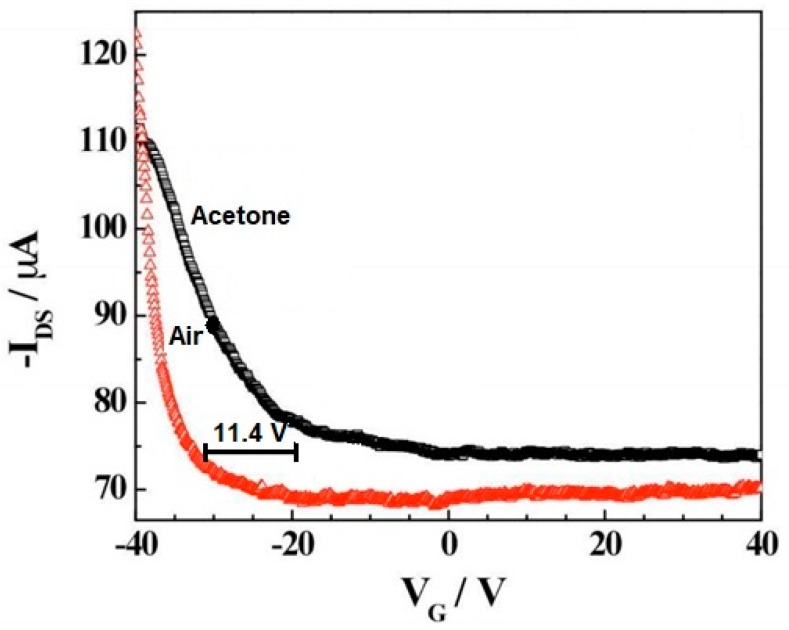
Transfer characteristic of iron tetraphenyl porphyrin-CNT in the presence of air and acetone. V_G_—gate voltage, I_DS_—the source-drain current. Reprinted with the authors’ permission [21]. The amount of voltage shift has been marked.

Figure 5 shows the field effect of the transistor transfer characteristic for the iron tetraphenyl porphyrin-functionalized CNT in the air and acetone vapor. A negative shift in 11.4 V in the gate voltage in the presence of air was observed upon exposure to saturated acetone vapors. This means a carrier concentration change in acetone environment compared to air. Simultaneously, a significant change in the iron tetraphenyl porphyrin-CNT mobility upon exposure to acetone compared to air can be noticed. This change in mobility indicates a decrease in the work function of the device on the absorption of analytes causing Schottky barrier modulation. A threshold voltage shift and mobility change in the case of CNTs-iron tetraphenylporphyrin hybrid suggest that the sensing mechanism has presided over the combination of electrostatic gating and the Schottky barrier effect [21].

**Figure 6:**
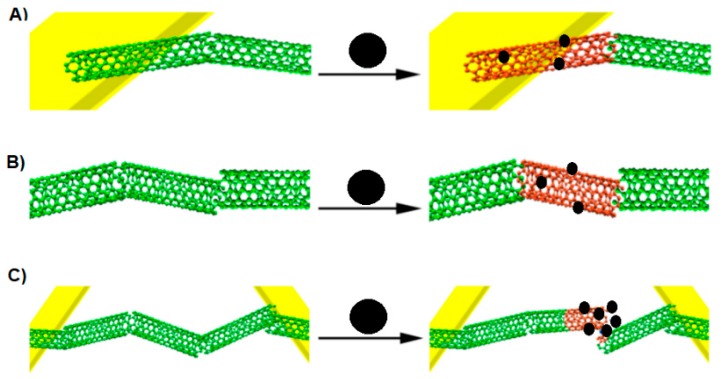
Three ways of CNT conductance change by analytes. (**A**) Modulation of the Schottky barrier at CNT-electrode junctions, (**B**) charge transfer between the analyte and CNT, (**C**) increasing the CNT–CNT junction distance by intercalation of the CNT network. Adapted from [26,29]. Black sphere- analyte, green cloud—current allowed, red cloud—current inhibited.

A chemiresistor built from a CNT network spanning two metallic electrodes has a current flowing through it when a voltage is applied. In the presence of an analyte, the current flow can be inhibited according to the following effects (Figure 6): modulation of the Schottky barrier at electrode-CNT junctions, charge transfer between CNT and the analytes, and increases in the CNT–CNT junction distance [26]. During the analysis, the conductance between two electrodes is measured to investigate a sensing response on an analyte. It is known that CNTs are composed almost entirely of surface atoms, therefore even a very small change in the environment will cause a change in the conductance. If the analyte is absorbed on the CNT-metal interface then the conductance may change by modifying the Schottky barrier. With regard to the fact that CNTs are not long enough to form channels, the conducting channels are formed by the connection of many CNTs. If the analyte is absorbed on the intertube junction, the conductance may change as well by disturbing of intertube junctions [26]. 

The analysis carried out by Calbi [27] proved that for some gas molecules the electric capacity of the grooves in the CNTs is inversely proportional to the length of the adsorbed molecule, while the internal capacity of the CNT depends inversely on the volume occupied by the molecule. In the case of some molecules, although the opening of nanotubes can increase the adsorption rate at entry into the channels, adsorption in external CNT grooves is much faster than in internal channels. As the external surface of the CNT is directly exposed to gas molecules, the adsorption process occurs in places outside and then through the diffusion, the molecules adsorb the CNT inside [28].

Mechanism of gas detection can rely on changing the material conductivity which is a result of the reaction of the gas molecule with the CNT surface. During the detection analysis of volatile compounds by e-noses, a conductivity measurement between two electrodes is performed. Due to the fact that CNTs are formed almost entirely from surface atoms, a small change in the chemical environment of CNT leads to a measurable change in conductivity. One of the reasons for the change in the sensor’s material conductivity is the change of Schottky’s potential barrier which occurs at the interface of metal and semiconductor at room temperature, i.e., on the electrode connector with CNT. At a higher temperature, e.g., 150 °C, gas molecules are adsorbed on the CNT wall and the charge transfer from the adsorbed gas to the CNTs contributes to the rise in the signal due to a change in conductivity. As a result of gas adsorption, electrons are transferred to the CNT conduction band that leads to a reduction in hole concentration and an increase in conductivity.

In a paper published by Peng, a change of the charge transfer in CNT exposed to NO_2_ and NH_3_ was observed [30]. It was shown that both CNT channels and CNT-metal connectors (Au electrode) play a pivotal role in the detection process. Control of the detection mechanism in the channels and on the CNT-metal connectors required isolation of individual elements from the gas by using a thin Si_3_N_4_ film. After isolating the CNT-Au connector and sensor exposure to NH_3_ no response from the CNT channels, even at high gas concentrations (above 500 ppm), was noted. However, after isolation of the CNT channels, the CNT-Au exposed to NH_3_ was highly sensitive at room temperature. The presented experiment suggests that NH_3_ induces Schottky barrier modulation which may be the predominant mechanism of NH_3_ detection by carbon nanotubes at room temperature. At temperatures above 150 °C, NH_3_ may adsorb to the CNT wall and play a role of an electron donor, whereas the Fermi CNT level moves towards the conduction band and the voltage threshold reaches a lower value. It should be also emphasized that NH_3_ reluctantly reacts with CNT in the pure form due to the significant activation barrier and preferential adsorption in structural defects. Furthermore, the activation barrier of NH_3_ adsorbed in CNT defects can be lowered by the presence of previously dispersed oxygen atoms. 

**Figure 7:**
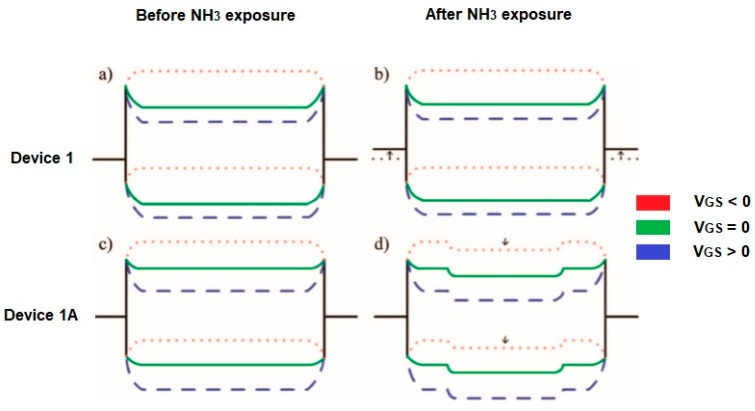
Scheme of the energy band diagram for device 1 (**a**) before and (**b**) after exposure on NH_3_. An intrinsic CNT is considered (the work function of the source/drain electrodes is initially near the valence band edge of the CNT) and for device 1A (the contacts passivated by Si_3_N_4_, the work function of electrodes aligns near the midgap of CNT. The Fermi level of the CNT channel shifts upwards due to electron-doping from NH_3_) (**c**) before and (**d**) after exposure to NH_3_. V_GS_- gate voltage. Partially reprinted with the authors’ permission [30].

A comparison of the two detection mechanisms related to the modulation of the Schottky barrier (SB) and the charge transfer (CT) shows some differences. For example, at room temperature, the absorption of NH_3_ on the CNT wall does not induce any effect and the sensing signal arises from the CNT/metal contact. When NH_3_ is absorbed on the CNT/metal interface then the electrostatic charge balance between CNT and metal is disturbed by NH_3_ dipoles, which results in an increase of the Schottky barrier for hole injection. The sensitivity is gate voltage-dependent. In Figure 7b, it is illustrated that a negative gate voltage (V_GS_) bending the energy band of the CNT upward leads to narrowing of the Schottky barrier width, holes can tunnel through the barrier. At a positive gate voltage, the Schottky barrier width is too small for tunneling. Using CNT channels as a sensing element, the charge transfer leads to moving the Fermi level of the exposed CNT channel upwards and the energy band shifts downwards. Thus, a potential barrier is created impeding the current flow (Figure 7d). Here, the charge transfer effect can be considered when the contact is fully protected. If not, with an increase of temperature, the sensitivity enhancement from the charge transfer, and the degradation of the Schottky barrier modulation counteract each other [30,31].

The SB detection is characterized by a very high sensitivity at room temperature but low reversibility, whereas the CT mechanism shows a low level of sensitivity at >150 °C and a high degree of recovery. The change of nanomaterials conductivity can be also connected to the reduction of the charge mobility in the CNT by forming areas disturbing the flow of charge. As we have already mentioned a single CNT does not have sufficient length to form conductive channels which are basically formed by combining multiple CNTs. Thus, if the analyzed substance is adsorbed in the connections between the nanotubes, the conductive channels are defective and the CNT conductivity will be modified [26]. In the case of a hybrid formed from carbon nanotubes (multi-walled CNTs) and SnO_2_, an observed signal amplification effect can result from the formation of CNTs embedded in SnO_2_. The formation of channels on the metal oxide surface may lead to an increase in gas molecules diffusion to the MOX surface, as well as to locally increase the electric field at the CNT-SnO_2_ interface [11].

The various sensing mechanisms of CNT-MOX (e.g., SnO_2_) were proposed. Wei et al. explain the gas-sensing by amplification effect of the PN junction structure between n-SnO_2_ and p-SWCNT [11]. However, Liu suggests the oriented growth of MOX along the CNTs during heat treatment [32]. As a consequence, the local electric field favorable for the gas-sensing reaction is improved. According to Wisitsoraat, the sensing mechanism is connected with an increase in the surface area due to the formation of CNT protrusions [33]. The authors of study [11] propose that the enhancement effect is attributed to the nanochannels formed by CNTs embedded in MOX. The formation of the nanochannels in the MOX surface can increase the diffusion of the gas molecules into the metal oxide surface as well as enhance the local electric field at the CNT–MOX interface. The effect of CNTs on gas-sensing is mainly on the surface, therefore the gas-sensing response is not dependent on the thickness in the case of the large thickness. The increasing surface area due to CNT’s intrusion and smaller grain size due to CNT doping can contribute to enhancing the gas reaction [11].

### 2.2. Sensors Based on Porphyrins and Hybrids

Porphyrins, complexed with metals, show greater sensitivity and selectivity than porphyrins in the basic form [34]. According to Capuano et al. an increase of the selectivity of metal complexes of porphyrins is most likely related to observed stabilization of the electronic system [34]. Our previous research show that the distribution of electrons, as well as aromaticity of the selected ligands with potential biological activity (i.e., antioxidant, cytostatic, antibacterial), may be stabilized particularly by metals characterized by high ionic potential (the ratio of electric charge to the radius of an ion) like Fe(III), Cr(III), La(III), Y(III), AL(III), and other 3d and 4f transition metals [35,36,37].

**Figure 8:**
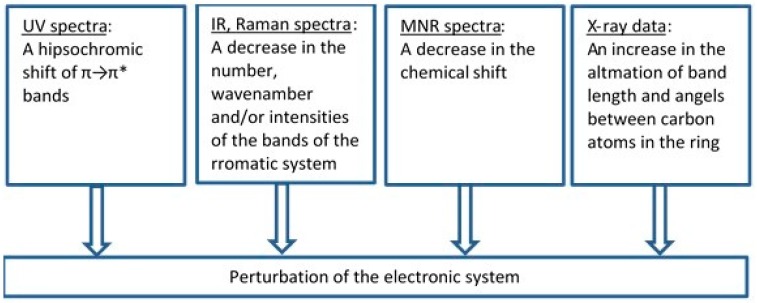
The criteria that were applied to estimate the degree of the electronic charge perturbation of molecule.

**Figure 9:**
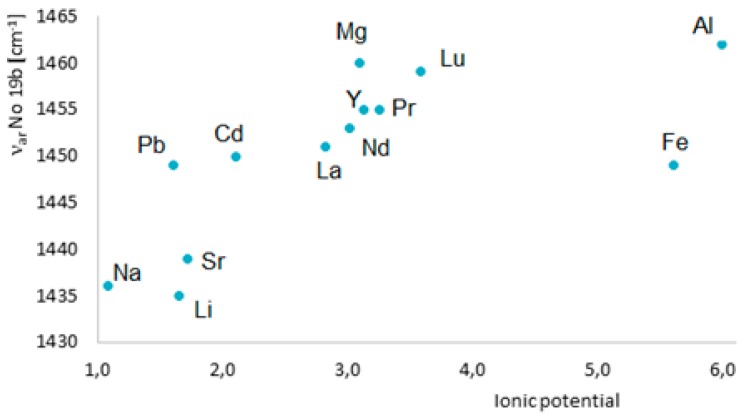
Dependence of the frequency of ν_ar_ band 19b in the Raman spectra of benzoates on the metal ionic potential [36].

To assess an influence of metals on the electronic system of different (aromatic) ligands with potential biological activity, we applied different methods such as the Fourier-transform infrared spectroscopy (FTIR), Raman spectroscopy (UV/VIS), nuclear magnetic resonance spectroscopy (^1^H and ^13^C NMR), X-ray diffraction, computational methods (based on the density functional theory, DFT), and aromaticity indices analysis. Figure 8 highlights the main spectroscopic criteria of stabilization or disturbance of the electronic system. Closer inspection of the electronic absorption spectrum (UV/VIS) obtained for ligands complexed with high ionic potential metals reveals that absorption bands related to π→π* transitions move to the longer wavelength (bathochromic shifts) which indicates that the electronic system is being stabilized. The analysis of a molecular rotational spectrum (FTIR and FT-Raman) reveals that bands characteristic for an aromatic moiety (e.g., bands at 160, 1590, 1500, and 1450 cm^−1^) marked by Versanyi [38] as 8a, 8b, 19a, and 19b, respectively, shift towards larger wave numbers or increase their intensity. An exemplary relationship between the aromatic moiety band 19b wave number increase and the ionic potential of metals is illustrated in Figure 9.

The opposite trends are observed for ligand complexes with low ionic potential metals such as Hg(I), Hg(II), Ag(I), Pb(II), and alkali metals. In this case, both, ligand’s electronic system and aromatic system are being disturbed. Moreover, bond polarization increases. Absorption bands related to π→π* transitions move to the shorter wavelength (hypochromic shifts). FTIR and FT-Raman bands characteristic for an aromatic moiety shift towards smaller wave numbers and their intensity decreases or vanishes. Here, X-ray data and aromaticity indices indicate differentiation of both, the length of bonds and angles between bonds in aromatic rings. Moreover, the delocalization of the electronic charge is decreased. We observed similar effects while analyzing oxygen adducts of Fe, Ru, and Os complexes with porphyrins by means of Raman spectroscopy and matrix isolation spectroscopy [39].

**Figure 10:**
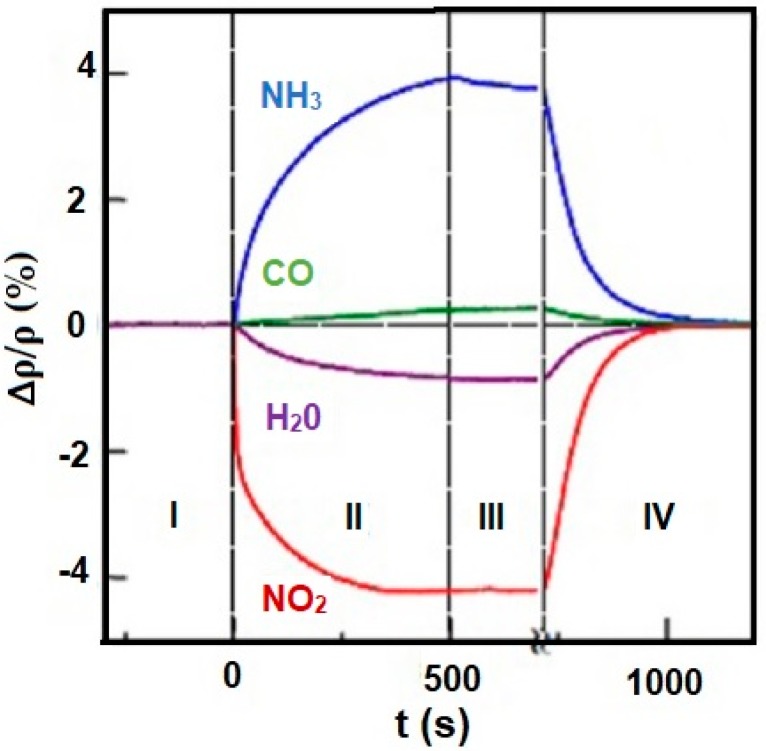
Changes in resistivity (ρ) of graphene exposed to NH_3_, CO, H_2_O, and NO_2_. Region I—the device in a vacuum before its exposure, II—exposure to a diluted chemical, III—the erasure of the experimental setup, IV—annealing (150 °C). Reprinted with permission [41].

Distribution of the electronic charge, as well as HOMO/LUMO (highest occupied molecular orbital/ lowest unoccupied molecular orbital), level occupancy determines not only reactivity and stability of molecules but also aromatic ring susceptibility to electrophilic substitution. An increase or decrease of an electronic density in the aromatic ring (including graphene and porphyrins rings) triggered by the influence of different metals, substituents, or even trace amounts of gases may result in changes in conductivity (resistivity) of electronic nose sensors. Figure 10 shows changes in resistivity ρ of graphene caused by exposure to various gases in the concentration of 1 ppm. Observed changes in the resistivity curve reflect the type of the compounds (electron donor or acceptor). Absorption of NO_2_ and H_2_O results in a decrease in resistivity which claims their electron acceptor nature, while absorption of NH_3_ and CO leads to an increase in resistivity which indicated their electron donor character [40,41].

**Figure 11:**
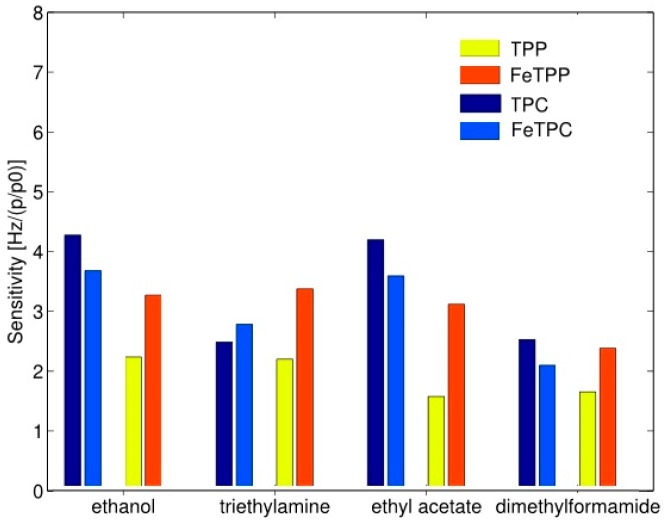
Sensitivity of free-base porphyrin (TPP) and porphyrin with iron (FeTPP) in comparison to analog porphyrin (TPC) and analog porphyrin with iron (FeTPC). Units of Hz per relative concentration (*p/p_0_*). Partially reprinted with permission [34].

The probable cause of an increased sensitivity of metal complexed porphyrins is the stabilization of its electronic system. It has been shown that there is a relationship between an increase of sensitivity and selectivity and the polar nature of volatile compounds. It suggests that the polar groups bind through hydrogen bonds in porphyrin analogs rings in which three hydrogen atoms are present instead of one. In the presence of polar compounds, an introduction of metal ions in the porphyrin analogs is not a sufficient method to improve the detection properties compared to the porphyrins in the basic form. Complexation with iron and magnesium always increases the sensitivity of porphyrins however, in the case of porphyrin analogs (*Corroles*), this effect was observed for magnesium whereas for iron it is not evident [34]. Figure 11 illustrates different behavior complexed metal ion. First, the sensitivity of basic form porphyrin analogs is larger than the sensitivity of porphyrin in the basic form. It is noticeable that the sensitivity of ethanol and ethyl acetate in the case of porphyrin analogs is more than doubled comparing to porphyrin. At the same time sensitivities, the other two compounds are almost identical. In the porphyrin case, the sensitivity of metal complexed porphyrin is larger towards all tested compounds than for free base porphyrin. In the instance of the porphyrin analog, it should be stressed that the inclusion of iron increases the sensitivity towards the compound for which the free base porphyrin analog has the lowest sensitivity and only in the case of triethylamine Fe complexed analog exceeds the basic form analog [34].

The structure of the CNT-metalloporphyrin hybrid sensor formed by the spraying of porphyrins on the CNTs film which forms specific aggregates was presented by Penza et al. [25]. Non-covalent interactions between CNT and porphyrins allow for a strong enough adhesion of metalloporphyrins to the surface of the CNT [8]. As expected, a significant increase in the sensitivity of the CNT-metalloporphyrin hybrid compared to pure CNTs was obtained. For example, the hybrid of CNT-metalloporphyrin with Mn in the porphyrin core exhibits 1.5-fold higher sensitivity to methanol at a concentration >10 ppm and 50% higher sensitivity to acetone and tetrahydrofuran compared to CNT.

The maximum sensitivity of the hybrid was achieved at low concentrations of methanol, acetone, tetahydrofuran, while the sensitivity decreased with the saturation of the environment with the analyzed substance. In the case of ethyl acetate, a significantly higher response using Zn-containing metalloporphyrins was achieved.

### 2.3. Sensors Based on Graphene and Graphene Oxides

**Figure 12:**
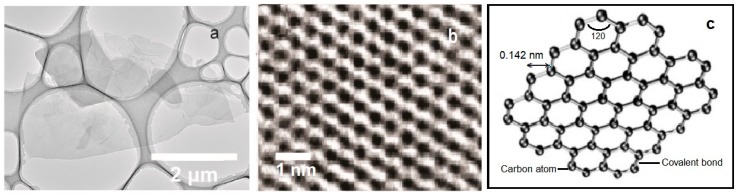
Morphological characterization of graphene structure. (**a**) Transmission electron microscopy image at low magnification showing several overlapping graphene flakes [43], (**b)** spherical aberration-corrected high-resolution transmission electron microscopy image of the honeycomb structure of a graphene single-layer sheet [44], (**c**) Single graphene nanosheet [45]. Partially reprinted with the permission of the authors [43,44,45].

Graphene (G), graphene oxides (GO), and reduced graphene oxides (RGO) are excellent materials for the construction of gas sensors due to their electronic, chemical, mechanical, and thermal properties, as well as high sensitivity to surface adsorption of gas molecules. The morphological characterization of the graphene structure was presented in Figure 12. It was observed that the graphene and reduced G oxides show sensitivity to trace amounts of NH_3_, NO_2_, H_2_O, Cl_2_, and CO [8]. The disadvantage of the e-nose constructed as a combination of graphene sensors is the difference in the signals from the sensors, despite the use of the same type of material for their production [8,42]. Differences in sensitivity of e-noses can be related to the size, thickness and random junctions between the flakes in various sensor devices. The difficulty of graphene-based e-nose detection may result from the use of a high number of sensors in the matrix (~20) to detect a small number of different gases (~4), as well as from the fact that the regeneration of sensors was challenging [42]. The differences among the signals of various graphene gas sensors can be connected with the random difference in gas sensitivities of sensor devices made from the same graphene materials. In spite of the use of the sensors constructed from the same graphene materials, the differences in shape, size, or thickness cause every single sensor to be different; this has an influence on e-nose sensitivity.

The electronic structure of graphene can be modified by gas molecules in diverse ways. CO_2_ and O_2_ adsorption convert the system to p-type semiconductor, while the NH_3_ adsorption leads to n-type behavior. The p- and n-type semiconducting behavior can be detected by applying and modulating gate voltage. Among all gas molecules considered, the absorption of the NH_3_ molecule can enhance conductance [46]. In the case of CO and NO molecules, the charge transfer towards graphene is observed, thus the conductivity of the sensor is increased [47]. NO_2_ can be an electron acceptor from the material where NO_2_ has been adsorbed, therefore, a decrease in electron density and an increase in graphene’s resistance is expected. According to Latif, similar conclusions were presented where the increase of graphene sensor resistance in contact with NO_2_ was shown [48]. The NO_2_ adsorption can take place in low energy centers of graphene via *sp*^2^ carbon atom or in high energy centers (oxygen groups and structural defects) [49]. NO_2_ is adsorbed mainly via oxygen functional groups [42]. As a result of the transfer of negative charge of oxygen functional groups to the NO_2_ molecule, the positively charged hole is formed in the graphene’s structure [42]. Thus, the presence of oxygen groups induces the disturbance of the graphene’s electronic structure. The effect can be enhanced by the ozonation of graphene which leads to the introduction of oxygen functional groups onto the surface of graphene which results in an increase of sensitivity towards NO_2_. The results of the experiment carried out by Nomani showed that the exposure of graphene to NO_2_ at a concentration of 500 ppb and 18 ppm caused a decrease in conductivity of 2.25% and 10%, respectively [50]. However, Jafri et al. presented a different point of view [51]. It was shown that the conductivity of graphene increases with the increase in the concentration of holes by more than one order of magnitude. This phenomenon is attributed to the generation of intermediate states in the area of defects which have properties similar to metals.

The adsorbed gases such as NO, NO_2_, NH_3_, H_2_O, H_2,_ and CO can act as electron donors or acceptors which may cause a change in the sensitivity and conductivity of graphene. Graphene, being a perfect crystalline material with a high surface to volume ratio, can undergo fluctuations in the charge carrier’s concentration by adding even several additional electrons. It is possible to distinguish two basic charge transfer mechanisms which lead to a change in graphene conductivity. Firstly, the charge transfer can depend on the relative position of the HOMO and LUMO orbitals. If the HOMO orbital is above the Fermi level of pure graphene (Dirac point) then the charge transfer takes place towards graphene. However, if the LUMO orbital is located below the Dirac point, then the charge transfer takes place towards the adsorbent particle. Secondly, the charge transfer between the absorbed molecule and graphene can be partially affected by the mixing of HOMO and LUMO orbitals with graphene orbitals (hybridization) [52].

According to the, well known, mechanisms the NH_3_ group which is an electron donor to the graphene aromatic ring leads to an increase in the electronic density in the ring and activates it by directing the substituent to the *ortho*- or *para*-position. Thus, electron donor groups improve the conductivity of graphene. NO_2_ as an electron acceptor that directs the substituent to the *meta*-position withdraws electrons from the graphene aromatic ring and thereby, inhibits electrophilic substitutions [53]. Electrophilic substitutions of type I (e.g., substituents: −NH_2_, −CH_3_, −C_2_H_5,_ activating the benzene ring) and type II (e.g., substituents: −NO_2_, −COOH, −SO_3_H, deactivating the benzene ring) can be used in the classification of trace amounts of gases. The type I substituents push the electrons into the ring and activate it, while the type II substituents pull out the electrons from the ring and deactivate it.

### 2.4. Graphene and Hybrid Sensors

An improvement of the sensitivity of the sensor by surface modification of reduced graphene oxides using various types of ionic liquids (IL) with a tailored structure was proposed by Zhu et al. [54]. It was shown that the ionic liquids have the ability to change the RGO semiconductor properties leading to a change in the type of conductivity from p-conductivity to n-conductivity. Therefore, it is possible to obtain the characteristic sensor response both for inorganic gases and organic odor molecules which allows us to distinguish them unequivocally. It has been experimentally demonstrated that RGO manifests a weak response to air and toluene and a good response to NO_2_ and Cl_2_. For comparison, RGO-IL shows a strong response to all four gases and a negative type of conductivity (type n). In addition, it has been proven that IL content has a significant effect on the conductivity of the gas-sensitive material, and the sensitivity of RGO increases with the increase in IL concentration. The mechanism of changing the RGO conductivity can be based on the presence of oxygen groups that lead to disturbance of the electronic structure. 

Alizadeh et al. suggest the significant differences in the selectivity of the sensor formed from RGO which depends on the conditions of oxide synthesis [8]. The formation of six types of RGO was obtained by experimental means by reducing two types of GO by hydrazine hydrate, ascorbic acid, and sodium borohydride hydrate. The oxidation process affects the intensity of exfoliation, the size of graphene oxide layers and the content of oxygen functional groups. Thus, the number of defects in GO is altered. The presence of oxygen groups leads to the extremely low conductivity of GO. However, it should be highlighted that the GO reduction can generate vacancies and structural defects which play a role in gas adsorption sites [55].

Some molecules with reducing properties can change the electronic and adsorptive properties of RGO by transferring oxygen, nitrogen or the entire functional group to the graphene structure. In the case of GO exposure to hydrazine, the oxygen functional groups are removed from the oxide, while the hydrazine nitrogen atoms are covalently attached to the GO surface. The nitrogen atom can play a function of the n-type dopant and modify properties of the final graphene product [56]. 

Sodium borohydride is a better reducing agent than hydrazine but introduces several heteroatoms to RGO. It has been demonstrated that NaBH_4_ effectively reduces carbonyl groups, however, it has low efficiency in reducing epoxides and carboxylic acids. Ascorbic acid is a moderate reducing agent compared to the previous two. The advantage of using vitamin C as a reducer is to minimize the risk of introducing heteroatoms into the RGO structure [57].

Results presented by Alizadeh indicate a significant effect of graphene oxidation and reduction of graphene oxides on the final porosity and surface of the obtained materials [8]. In the case of the two-stages graphene oxidation process, a greater number of defects and oxygen functional groups are generated in the GO structure than in the case of single-stage oxidation. In the same paper, the influence of the graphene sensor synthesis on gas detection was also described. For this purpose, analysis of detection parameters was carried out for 10 ppm of dichloromethane, ethanol, benzene, toluene, acetone, diethyl ether, and n-hexane. Graphene was reduced using two reducing agents (hydrazine and ascorbic acid). Depending on synthesis conditions, there were differences in response to individual gases, especially differences in the ΔR/R_0_ parameter, where ΔR is the resistance difference between the sensor response after and before the odor exposure, and R_0_ is the resistance due to the response on the odor exposure. Depending on the reductor used, the difference in the ΔR/R0 parameter was 3% for ethanol and 5% for acetone [8].

Experimental and theoretical studies proved that the sensitivity of graphene-based sensors can be significantly improved by doping with Br, N, P, Ga, Cr, Mg, S, and Si. Doping leads to the formation of new active sites on the graphene’s surface which have an ability to strongly adsorb gas molecules. For example, doping graphene with Mg and Cr results in an increase in sensitivity towards SO_2_ [58,59], while doping graphene with Fe, N, and N and Si combined improves sensitivity towards H_2_S, CO, and NO_2_ [60,61], respectively. In the case of N and Si doping, the N atom is the active site of NO_2_ adsorption, while doping graphene with Si significantly improves the sensitivity to NO and NO_2_. The sensor built of N-Si-G shows sensitivity towards 21 ppm NO_2_ and the sensor response declines along with the gas concentration to ~1 ppm [62]. In addition, the introduction of defects in the graphene structure by doping with Br, S and N results in improved sensitivity towards formaldehyde [63]. The summary of hybrid materials, graphene, and carbon nanotube parameters and the detection limit of selected gases are presented in Table 1.

## 3. The Proposed Interpretation of the Sensing Mechanism based on Influence the Metals and Substituents on the Electronic System

To enhance the interpretation of the sensor detection molecular mechanisms in the e-nose, we used our previous analysis of the influence of metals and substituents on the electronic systems of carbon and heterocyclic rings of selected aromatic ligands [35]. We showed that transition metals with high ionic potential and delocalized orbitals (especially 3d, 4f) such as Fe(III), Cr(III), Mn(II and III), Zn(II), Ln(III), Al(III), and Mg(II) stabilize the electronic systems of ligands and delocalize electronic structure. The stabilization of electronic systems and structure delocalization during complexation of ligands with metals has been observed in carbon rings, heterocyclic aromatic acids [35,36,37], and porphyrins [39]. Substituents with high ionic potential such as F, Cl, and NH_3_ (including the type I substituents) increase the electronic density in the aromatic ring and stabilize electronic systems. Substituents with low ionic potential (e.g., iodine) destabilize the electronic systems of aromatic ligands [35,39,85,86]. The literature data indicate that gases such as NO_2_, CO, SO_2_, CO_2_, N_2_O_3_, and NO radically reduce the conductivity of graphene, while NH_3_ increases its conductivity. In addition, metal-complexed porphyrins (Fe, Zn, Mn, Mg, Al) shows greater sensitivity and selectivity to trace amounts of NH_3_ than porphyrins in the basic form.

The comparison of our previous research with the analysis in the literature suggests that the increase of graphene electronic density (e.g., under the exposure of NH_3_) and delocalization of the electronic charge distribution results in an increase of the graphene sensor conductivity. Both phenomena show a certain analogy to the metal’s electrical conductivity. High electronic density and delocalized charge surrounding the atomic strands of metals are the cause of the excellent conductivity of these substances. The presented idea of the radical change of the graphene conductivity under the influence of type I and II substituents and the change of sensitivity and selectivity of porphyrins complexed with metals is at this stage purely qualitative. The quantitative confirmation of the described relationships requires further research.

The interpretation we have proposed concerns sensors composed of porphyrins and metal complexed porphyrins as well as the sensors composed of pure graphene. It should be stressed that the molecular mechanisms in the case of graphene oxide are more complicated which was confirmed by literature data [48,49,87,88,89,90,91]. It should be emphasized that each substituent/atom (connected by a single or double bond) and incorporant affect the structure of the σ- and π-electrons of the primary system (if the π-electron structure is formed). The measure of these interactions can be the EDA descriptors (electron donor-acceptor, which was established within the framework of the natural bond orbital (NBO) theory). The sEDA and pEDA descriptors show the number of electrons shifted to or withdrawn from the σ- and π-valence orbitals of the core molecule which has been incorporated or substituted (through a single or double bond) by heteroatom or heteroatomic group. It can be noticed that the influence of selected substituents/incorporants on the electronic structure is not obvious and unambiguous because, e.g., the substituents such as −NO_2_ and −NH_2_ withdraw σ-electrons from the system. At the same time, −NO_2_ withdraws π-electrons, and −NH_2_ donates π-electrons to the system [90]. In the case of incorporation of −O− (oxygen atom incorporated in monocyclic systems), the effect of withdrawing σ-electrons from the system and the effect of donating π-electrons can be observed [81]. In the case of the =O (an oxygen atom connected by a double bond), the effect of withdrawing both σ- and π-electrons from the system can be observed. The influence of double-bonded substituents like =NH and =NO on an electronic system is similar [89].

The molecular mechanism, which was proposed by us assumes that a radical change in graphene conductivity in the presence of traces of various gases should also refer to the theory of semiconductivity. Schottky’s barrier and the energy gap separating electron states: occupied from the valence band and empty from the conduction band are of great importance for the phenomena considered here [92]. If this energy gap is significant then the material is an insulator. At very low temperatures, around 0 K this material quite inhibits the flow of electric current. When the energy gap is nonzero but still small (conventionally up to 4 eV), this material acquires semiconductor properties. Depending on the conditions, graphene may exhibit semimetal characteristics, but its characteristics strongly depend on various factors. According to Schottky and Horowitz, the two distinct cases can be separated, depending on injection barrier height: poor injection (high-barrier) and effective injection (low-barrier). In the former case, the organic semiconductor behaves as a perfect insulator [92]. Nanotubes have highly delocalized and extended π-electron systems and, depending on the chirality, can be semiconductors, the same as metals. Adsorption of molecules of various gases or liquids on its surface, intermolecular interactions, and acceptor-donor effects affecting the distribution of electron charge in molecules have a special impact. Our interpretation also indirectly supported by various literature data [92,93], assumes that porphyrins can affect the energy gap and Schottky’s barrier size in semiconductors, facilitating the transfer of electrons from the valence band to the conduction band. This phenomenon can be compared to the effect of a positive catalyst on the rate of chemical reaction, which reduces the activation energy (barrier)—in the case of graphene, the reaction associated with the transfer of electrons to the conductivity band. The question arises why porphyrins (and their complexes with some metals) increase graphene conductivity. According to our research [35,36,37,39] due to several aromatic rings, porphyrins have highly delocalized electrons—an electron cloud capable of rapid movement as in metals. Complexing with metals (especially those with high ionic potential, such as Fe(III), Cr(III), Ln(III), and Y(III)) increases electron delocalization, giving some aromatic compounds super-aromatic properties. As mentioned above, examples are ferrocene and dibenzenochrome. Our data partly confirm the works of Langa et al. [93], which showed that porphyrins, graphene, and carbon nanotubes have been found to be excellent building blocks for the construction of donor-acceptor systems. To sum up, the interaction of porphyrins and their complexes with metals in our opinion affects the delocalization of the electron charge in graphene and nanotubes, affects the energy barrier and the size of energy gaps between valence and conductivity bands. In consequence, the conductivity in graphene can change significantly.

## 4. Conclusions

The literature review clearly shows that the detailed detection mechanism of e-nose is not fully elucidated and requires further analysis using, among others, quantum mechanics, and molecular modeling methods. Our studies concerned the influence of substituents and metals on the stabilization or disturbance of the electronic system of aromatic ligands. We investigated the effect of metals and substituents at type I and II differing significantly in the ionic potential on the electronic structure of aromatic rings [35,36,37]. Our research and proposed conclusions can be helpful to understand the molecular mechanisms of a radical change (increase or decrease) of graphene conductivity in the presence of trace amounts of various gases. We suggest that an increase in the electronic density of graphene and an increase in the delocalization of the electronic charge increases the sensor conductivity. Incorporation of porphyrins with selected metals increases the sensitivity and selectivity of porphyrin sensors towards volatile compounds like methanol, ethanol, triethylamine, ethyl acetate, and dimethyloformamide. Sensors complexed with metals can be a good analogy to super aromatic compounds with a strongly delocalized electronic charge distribution and motile electrons. An increase of the electronic delocalization degree and electrons mobility shows that sensors can be similar to electronic structures found in pure metals.

In the case of graphene oxide, doped graphene molecular mechanisms suggest that an increase of the “electron holes” concentration in graphene leads to conductivity decreases. Not only graphene but also valence electrons localized on functional groups coordinated to the carbon ring can be a conductor. The subtle and sensitive balance in the electronic charge distribution between carbon rings and functional groups is maintained. If the delocalization and mobility of valence electrons are increased the electronic structure is similar to the structure of the metallic conductor.

Based on semiconductivity theory and Schottky’s barrier, the size of the energy gap separating electron states (occupied from the valence band and empty from the conductivity band) and the phenomena of electron transfer can significantly affect the change in graphene conductivity under the influence of trace amounts of various gases.

The proposed approach to the gas detection mechanism can open or facilitate the search for further practical applications of graphene, nanotubes, and porphyrin derivatives in various fields of science and industry. For the quantitative confirmation of the presented thesis, further experimental and theoretical studies are necessary.

## Figures and Tables

**Table 1:** A summary of properties along with an approximate detection limit for selected compounds, sensors based on graphene and its oxides, as well as hybrid materials of graphene and carbon nanotubes.

Type of Sensor	Identified Chemical Compounds	Approximate Detection Limit	Selectivity	Time Response and Responsivity
Hybrid CNT-SnO_2_ [11,22,23,24]	NO_2_	2 ppm	Ethanol, Methanol	~20 s, 2680 A/W [64]
	NH_3_	1 ppm		
	CO	0.5 ppm		
	O_3_	21 ppb		
	H_2_, CH_3_OH, C_2_H_5_OH	<100 ppm		
Hybrid SWNT-COOH-polymer [18,65]	NH_3_, NO_2_ (CH_3_CH_2_CH_2_)_2_NH, (CH_3_)_2_NH, N(CH_3_)_3_, NH_4_OH, Alcohols, Ketones, Aldehydes	100 ppm 50–1000 ppm	Ammonium hydroxide Acetic acids Acetone Ethanol	~7 min, 41–64 mA/W [66]
Hybrid SWNT-porphirine [67,68]	Alkanes, Amines, Aromatic hydrocarbons, Ketones, Alcohols, Formaldehydes, Nitrotoluene	5–2000 ppm	Alkanes, Amines, Ketones	60–80 s, 10^1^–10^2^ A/W [69]
G [40,48,70]	NO	160 ppq	Chloroform, Methanol, Tetrohydrofuran, Acetonitryle, Ethanol, Toluene, Methylene chloride	~11 s, >1 A/W [71]
	CO_2_	3 ppm		
	NO_2_	<200 ppb		
	NH_3_	1 ppm		
GO [72,73,74,75]	NH_3_	0.02 ppm	Acetone, H_2_S, CO, ethanol, methanol, NH_3_, NO_2_ Metal oxide	<15 s [71]
	NO_2_	~1 ppm		
	H_2_	20 ppm		
	CO	50 ppm		
	H_2_S	ppb level		
	CH_3_OH, C_2_H_5_OH	ppm level		
	C_3_H_6_O	ppm level		
RGO [72,73,76,77,78,79,80]	NH_3_, CO	10 ppm	Benzene, Acetone, Dichloromethane, Toluene, Ethanol, n-hexane NH_3_	~150 s ~18 min, 0.73 A/W [81]
	NO_2_	0.25 ppm		
	H_2_	200 ppm		
	H_2_S, NO	~5 ppb		
	CO_2_	20 ppm		
	CH_3_OH, C_2_H_5_OH,	~100 ppm		
	Benzene, Toluene	ppm level		
Hybrid G-polymer [46,82,83]	NH_3_, CO_2_ NO_2_	5 ppm 0.25 ppm	NH_3_, NO_2_ CO_2_ H_2_ H_2_S Ethanol	36 s–3 min 8 s 1 s–3 min 5-60 s 2–6 min, ~10^4^ A/W [84]
